# Challenges of Implementing Antenatal Ultrasound Screening in a Rural Study Site: A Case Study From the Democratic Republic of the Congo

**DOI:** 10.9745/GHSP-D-16-00191

**Published:** 2017-06-27

**Authors:** David Swanson, Adrien Lokangaka, Melissa Bauserman, Jonathan Swanson, Robert O Nathan, Antoinette Tshefu, Elizabeth M McClure, Carl L Bose, Ana Garces, Sarah Saleem, Elwyn Chomba, Fabian Esamai, Robert L Goldenberg

**Affiliations:** a University of Washington, Seattle, WA, USA.; b Kinshasa School of Public Heath, Kinshasa, Democratic Republic of the Congo.; c University of North Carolina at Chapel Hill, Chapel Hill, NC, USA.; d RTI International, Durham, NC, USA.; e Aga Khan University, Karachi, Pakistan.; f University of Zambia, Lusaka, Zambia.; g Moi University, Eldoret, Kenya.; h Columbia University, New York, NY, USA.

## Abstract

Persistent global disparities in maternal and neonatal outcomes and the emergence of compact ultrasound technology as an increasingly viable technology for low-resource settings provided the genesis of the First Look Ultrasound study. Initiated in 2014 in 5 low- and middle-income countries and completed in June 2016, the study's intervention included the training of health personnel to perform antenatal ultrasound screening and to refer women identified with high-risk pregnancies to hospitals for appropriate care. This article examines the challenges that arose in implementing the study, with a particular focus on the site in Equateur Province of the Democratic Republic of the Congo (DRC) where the challenges were greatest and the efforts to meet these challenges most illuminating. During the study period, we determined that with resources and dedicated staff, it was possible to leverage the infrastructure and implement ultrasound at antenatal care across a variety of remote sites, including rural DRC. However, numerous technical and logistical challenges had to be addressed including security of the equipment, electricity requirements, and integration of the intervention into the health system. To address security concerns, in most of the countries field sonographers were hired and dispatched each day with the equipment to the health centers. At the end of each day, the equipment was locked in a secure, central location. To obtain the required power source, the DRC health centers installed solar panels bolted on adjacent poles since the thatch roofs of the centers prohibited secure roof-top installation. To realize the full value of the ultrasound intervention, women screened with high-risk pregnancies had to seek a higher level of care at the referral hospital for a definitive diagnosis and appropriate care. While the study did provide guidance on referral and systems management to health center and hospital administration, the extent to which this resulted in the necessary structural changes varied depending on the motivation of the stakeholders. In order for such an intervention to be scaled up and sustained as part of a health system's general services, it would require considerable effort, political will, and financial and human resources. Preliminary results from the study indicate that taking routine antenatal ultrasound screening to scale is not warranted. Lessons learned in implementing the study, however, can help inform future studies or programs that are considering use of ultrasound or other imaging technology for other applications in low-resource settings.

## Abstract (precis)

In the context of a well-resourced research project on obstetric ultrasound, we encountered major challenges, including security and maintenance of the equipment, electricity requirements, health systems integration, and a variety of other systems issues. We propose future ultrasound interventions have at minimum a functioning health system with skilled and motivated staff, access to a referral hospital capable of providing affordable and higher levels of care, and feasible transportation means.

## BACKGROUND

### First Look Ultrasound Study

The Global Network First Look Ultrasound study was a cluster-randomized trial whose primary aim was to assess the impact of antenatal ultrasound screening performed by health care personnel on a composite outcome consisting of maternal mortality and maternal near-miss, stillbirth, and neonatal mortality in low-resource, rural community settings. The study addressed the lack of maternal and neonatal outcome data based on actual deployment of compact ultrasound in rural, low-income country settings brought to light in a seminal article by Harris and Marks.^1^ Details of the First Look study, including ethical reviews and approvals, are discussed in depth elsewhere.^2^

Briefly, the study used the existing research infrastructure of the Global Network for Women's and Children's Health Research sites in the Democratic Republic of Congo (DRC), Guatemala, Kenya, Pakistan, and Zambia. Central to each site was the development and maintenance of the Global Network Maternal Newborn Health Registry in defined geographic areas or clusters. Within these clusters, the Registry documented all pregnancies and their outcomes to 6 weeks post-delivery, providing population-based rates of stillbirth, maternal and neonatal mortality and morbidity, and health care utilization.^3^ In the 5 countries, the First Look study was conducted in 58 clusters (29 clusters were randomized to the intervention and 29 to the control). Clusters were defined geographical areas with at least 1 health center and approximately 500 births per year. The study began in 2014 and was implemented for at least 18 months in each country site; it was completed in all sites in June 2016.

The intervention included 4 components:

1. Training of ultrasound-naïve health workers (e.g., nurses, midwives, clinical officers) to perform 2 ultrasound screening examinations during antenatal care, targeted at 18–22 weeks gestation and at 32–36 weeks gestation
2. Training of referral hospital staff in emergency obstetric and neonatal care as necessary
3. Guidance to Ministry of Health officials and hospital administrators on possible referral system enhancements
4. Community sensitization activities to inform women and their families of the availability of ultrasound at their antenatal care clinics

The main focus of the intervention was on the first component—training health workers to perform ultrasound examinations and to refer high-risk pregnancies to a higher level of care. Trainees were taught to assess gestational age, to identify high-risk pregnancies (including multiple gestations, fetal anomalies, mal-presentation, placenta previa, and risk of preterm delivery), and when and how to refer to the hospital for appropriate care. A 10-day course was administered under the supervision of the University of Washington, Department of Radiology (UW), in the 5 Global Network country sites. In concert with local sonographer trainers, UW then oversaw a quality assurance process including review of stored images of all ultrasound scans collected during a 3-month pilot period and 10%–20% of images collected throughout the study. Both the initial training and the quality assurance process are discussed in detail elsewhere.^4^ In the DRC, Pakistan, and Zambia, the sonographers were based in the field and traveled to the health centers in their assigned clusters each day with the necessary equipment to perform ultrasound examinations. In Guatemala and Kenya, the sonographers and equipment remained stationed at the health centers. Control clusters had the Registry, but none of the 4 interventions described above.

The main focus of the First Look Ultrasound study was training health workers to perform antenatal ultrasound and to refer high-risk pregnancies to a higher level of care.

The purpose of this article is to examine the challenges arising during implementation of the study's ultrasound intervention, with a particular focus on the study site in the DRC where the challenges were the greatest.

## PERFORMANCE SITE IN THE DRC

While the study was being conceived, the Global Network was reestablishing the Maternal Newborn Health Registry in Equateur Province in the DRC. Equateur Province was also emerging from a period of political conflict and peace negotiations, lasting from 2009–2013. The study team conducted an initial assessment of the DRC site to determine whether the infrastructure in Equateur Province was sufficient for the study's ultrasound intervention. The Global Network's decision to conduct the study in Equateur Province was made with the understanding that its inclusion would examine the effectiveness of the ultrasound intervention across a broader range of resource-limited settings, with Equateur Province representing the lower boundary.

Equateur Province is located in northern DRC bordering the Republic of the Congo to the west and the Central African Republic to the north. Karawa, the town around which the study is centered, is situated 68 kilometers east of Gemena along National Highway 24—a red clay roadway maintained by hand and shovel—which can take 3 hours or more to travel by vehicle depending on the season. The Gemena airport had weekly 4-hour flights to Kinshasa via Mbandaka, a journey that might otherwise take 1–2 months by river and land. Houses in the area are made of wooden poles, earth, and thatch, as are most health centers, with the addition of plaster and paint. Farming is central to livelihood and networks of cropland and streams surround the villages, linked by paths sometimes wide enough for a vehicle to pass.

The Karawa Mission, where the hospital is located, has endured extended periods of evacuation during stretches of political conflict and war since 1960. The impact of periods of substantial support that brought running water, hydroelectricity, an airstrip, and significant infrastructure to the mission is apparent, as is the toll of decades of isolation. The hospital, consisting of buildings several decades old separated by manicured hedges, houses a maternity ward with 20 beds and an active operating room. During the trial period, electricity was generally available on demand from a generator, and water carried and stored in barrels. Five Congolese physicians led the hospital's staff.

## FEASIBILITY ASSESSMENT

Before exploring the challenges faced during implementation of the study in the DRC, we first review the inclusion of Karawa Hospital and its satellite health centers in the study to shed light on the criteria essential to making ultrasound feasible for research and implications for broader sustainability. To determine whether Karawa Hospital and its health centers would be suitable for the study, we first determined whether the intervention was appropriate for the health care environment. We then determined the extent to which providers and health care administrators were willing to participate in the study.

### Appropriateness of the Ultrasound Intervention in the DRC

We anticipated several critical features of the environment that would be necessary to realize any potential improvements in perinatal outcomes as a result of antenatal ultrasound screening. First, each health center in which ultrasound was performed had to have a power supply and adequate security for the ultrasound units. In addition, the surrounding health system needed to have basic functions, including health centers staffed with trained personnel providing antenatal care, guidance for referrals, and assistance in deliveries. The value of obstetric ultrasound screening is minimized when patients with high-risk pregnancies are unable to attain the level of care that screening indicates. Therefore, a referral hospital that provided continuous Comprehensive Emergency Obstetric and Neonatal Care (CEmONC) had to be available.^1^ Karawa Hospital partially met this requirement. Although some of the signal functions of CEmONC were not always readily available, including blood and anesthesia, the hospital did have a functioning operating room and physicians who perform cesarean deliveries.^5^ Blood was often supplied by relatives with matching blood types and anesthetics purchased in shops outside the hospital when necessary. However, provision of these resources requires additional time and planning and therefore may have limited the impact of the ultrasound intervention. Intermittent power precluded the hospital's ability to maintain a blood bank, but the generator could provide power to the operating room as needed.

The value of obstetric ultrasound screening is minimized when patients with high-risk pregnancies are unable to attain the higher level of care that screening indicates.

Another essential feature was the ability of patients to reach the referral facility. The availability of a vehicle for transport is ideal but not essential for the effectiveness of the intervention.^1^ Ultrasound screening can provide a woman with knowledge of a high-risk pregnancy, the time to plan, and encouragement to travel to the referral hospital prior to delivery. In Equateur Province, most likely a woman would need to walk to the hospital. Bicycles are sometimes available, but motorcycles are rare. Canopied bicycle trailers for transporting semi-recumbent patients are available at some health centers. There is a limit, however, to the distance a pregnant woman in late pregnancy or with an emergent problem can reasonably travel without advanced modes of transportation.

The cost of hospital care was also an important consideration. An alternative hospital with its surrounding health centers was considered as a study site; however, the cost of a cesarean delivery at this hospital was the equivalent of US$50 and the cost of a vaginal delivery, approximately US$10. These prices represent an enormous barrier to care for a population living largely beneath the poverty line, with limited access to money. The catchment area for Karawa Hospital, on the other hand, was supported by a 5-year grant from the United Kingdom's Department for International Development (DFID), which lowered these costs by 80% throughout the study period.^6^ While this certainly limits the scalability of the intervention in Equateur Province beyond the limits of the DFID grant, it was more closely aligned with other Global Network sites, such as Kenya and Zambia, where maternal health care is ostensibly free. The DFID grant also supported the hospital and health centers with medicines and supplies, increasing the value of attending antenatal visits.^7^

### Willingness of Health System Management and Staff

Two recent studies have emphasized the importance of having willing providers to implement ultrasound interventions in low-resource settings.^8^^,^^9^ Of similar importance is the enthusiasm and participation of those managing health systems, because improvements must be made to take advantage of the new information made available by ultrasound screenings. As such, the commitment of potential referral hospitals and zonal health systems to employ the intervention was factored into selection of the clusters. The health system in the Karawa area demonstrated such commitment, further differentiating it from other health systems assessed. Karawa Hospital had a physician with ultrasound experience and a keen interest in the study's intervention. This contrasted with the indifference and inquiries about compensation expressed at the other potential hospitals. Similarly, the zonal health coordinator in Karawa was eager to undertake the study.

## CHALLENGES TO IMPLEMENTING THE ULTRASOUND STUDY

Many of the challenges faced in the study and the actions taken to meet them reflect the temporary nature of the study and the innovative nature of the intervention. Addressing these challenges required balancing the desire to simulate an intervention that could be studied and potentially taken to scale with constraints of time and limits of influence intrinsic to the study's design. In some cases, the choices were straightforward. For example, at the outset, requests came from each health system hosting the study for transportation to assist women requiring a higher level of care. We did not provide or subsidize transportation because we believed that to do so would confound the study results and impact inferences about scalability.

Ultimately, the specifics of the study implementation at each site were the result of negotiated agreements of a broad partnership of donors, academic institutions, and research organizations. Each Global Network country site is co-managed by investigators from a local and a U.S.-based university, each has a large degree of autonomy, and each has developed longstanding relationships with the health systems that encompassed their clusters. The choices made to meet the challenges of implementing the study reflect a process that incorporates all these points of view. Below is a discussion of the challenges faced and how they were addressed, with an eye toward guiding similar interventions going forward.

### Security

The security of the ultrasound equipment was a substantial concern from the conception of the study. The GE Healthcare Logiq-e ultrasound unit, including console, transducer, printer, and accessories, sold for more than US$22,000 at the beginning of the study. The stark contrast of this to the value of existing equipment in targeted health centers necessitated that extraordinary measures be taken.

In the case of the DRC site, the ultrasound machines were locked up each night at a staff member's house within the Karawa Mission premises, and the trained field sonographers transported them to the health centers daily by motorcycle. At some other sites, the distance of the health centers from a secure, central storage location made this approach impractical. In Kenya, for example, the equipment was locked each evening in custom-built metal cabinets that were securely bolted to the walls of rooms with lockable doors, within health centers guarded by security personnel.

At the DRC site, field sonographers transported the ultrasound equipment to the health centers daily by motorcycle and locked the equipment each night at a staff member's house.

That these measures are not likely to be replicable on a large scale may be balanced by the understanding that the GE Healthcare Logiq-e is substantially more technologically robust than its use in the study warranted. This, coupled with the continuing reduction in the price of ultrasound technology,^1^ may obviate the need for these measures over time. However, similar programs will need to consider the security of equipment.

### Electricity

In initial assessments of the health facilities in Equateur Province, none of the health centers had electricity. In some health centers, solar power had once been available, but the solar panels, inverters, and/or batteries were missing at the time of our visit. We were informed that the equipment had either been stolen or removed for the purpose of security. The initial assumption was that the source of power for the ultrasound devices would need to be a portable generator brought daily to the health center along with the ultrasound machine. DRC site staff, however, opted to install solar panels bolted on 10-foot poles next to the health centers. The thatch roofs of the health centers prohibited secure roof-top installation. Batteries and inverters were secured in locked boxes within the health centers. Each health center has security personnel to protect medical supplies and equipment, and the combination proved sufficient during the trial.

Electricity needs of the ultrasound equipment were met in the DRC by installing solar panels bolted on poles adjacent to the health centers.

In Pakistan, large batteries, or uninterruptible power supplies (UPSs), were charged throughout the night at the site office and transported along with the ultrasound machines to health centers daily. In Kenya and Zambia, the health centers are connected to the electrical grid, although power has become increasingly intermittent. The GE Healthcare Logiq-e ultrasound came equipped with 2 additional batteries, each with an initial working time of 45 minutes. These often sufficed during power outages, though Kenya also employed UPSs.

Electrical power demands of the Logic-e ultrasound are greater than the newer tablet-size versions that are now available. The corresponding reduction of the size and cost of solar equipment should simplify what is required to power ultrasound technology, but electricity requirements remain an important consideration in the implementation of this type of intervention.

### Ultrasound Machine Servicing

The challenge of servicing the ultrasound machines in Equateur Province was anticipated, and 2 spare ultrasounds were supplied by GE Healthcare. When a machine malfunctioned, the unit had to be shipped back to Kinshasa, where a service provider contracted by GE Healthcare managed repairs. This process took 3 months or more, in large part due to the complexities of importing replacement parts. The Office of the United States Trade Representative describes the DRC as having “complex regulations, a multiplicity of overlapping administrative agencies and a frequent lack of professionalism and control by officials responsible for the regulatory environment.”^10^ Attaining exemptions for charitable donations in this environment in a reasonable amount of time was not possible. As a result, the costs of importing the replacement parts could equal the cost of the parts themselves. At the other country sites, GE Healthcare had established relationships with local service providers prior to the study, and the repair process was less time consuming and expensive. For more permanent ultrasound interventions, greater emphasis on developing and costing the management of maintenance at the outset appears warranted.^11^

### Supply Chain

An appealing aspect of the ultrasound intervention was the limited amount of supplies required, which consisted only of gel and paper towels for cleaning the gel off of patients. The study also supplied printers, with the thought that providing an ultrasound image to patients may encourage family members to support attendance at antenatal visits and compliance with recommendations for referral. The printers required thermal paper. Both the gel and the paper had to be shipped from Kinshasa to Equateur Province. This required planning and bulk purchasing, neither of which was a problem during the study.

### Field Sonographers

In Equateur Province, nurses other than those stationed at health centers were trained to perform ultrasound screening for the study in part due to logistical considerations. Hiring nurses both to transport the ultrasounds by motorcycle and to screen patients addressed the practical matter of transporting 5 ultrasound units from Karawa Mission out to remote health centers and back each day. Similar considerations led to the hiring of external medical personnel as field sonographers in Pakistan and Zambia.

Another factor that influenced the decision to hire nurses outside of health centers to perform ultrasounds for the study relates to the limit of influence the study could have on health systems. While the study aimed to simulate an environment in which health center personnel integrated ultrasound screening into antenatal care provision, the extent to which nurses and midwives stationed at health centers would engage in screening without active supervision was a concern highlighted in another study.^8^ A health system's willingness to participate in the study was not a guarantee that it could, for 18 months, muster the managerial capacity and will to ensure compliance to screening at the health center. The additional time and effort required of nurses to collect data for each screened patient was also a concern specific to the study. These considerations led to an approach at the Kenya site—where the ultrasound equipment was stored at health centers—of hiring, training, and stationing clinical officers at those health centers. In Guatemala, nurses and medical officers employed by the Ministry of Health and permanently stationed at health centers were trained and engaged for the study, though some needed assistance by site-employed field sonographers to ensure that sufficient screenings occurred. Engaging existing health center personnel for this intervention required structural change within the health system, which was beyond what could be orchestrated for a limited period by external researchers, but important for future endeavors.

Engaging health center staff for the intervention required structural change within the health system that was beyond the scope of the research project, so we hired field sonographers instead.

### Training

The preference of the study was for training to take place in the hospitals where study patients would be referred during the trial. This created the potential for hospital sonographers to be engaged as co-trainers or participants, depending on their level of skill. By doing so, the hospital could be made aware of the intervention, and the hospital sonographers and trainees could develop relationships, which may have enhanced communication about referrals. This was possible in Karawa Hospital with the inclusion of a doctor and nurse from the hospital. Because the hospital was substantially smaller than those where trainings took place in other country sites, there was a concern about having a sufficient number of patients to screen during the training. The goal was to have each trainee complete at least 2 scans each day during the 10-day course. Each woman, for the sake of comfort, could only be scanned once. This amounted to having at least 16 women with second- and third-trimester pregnancies at the hospital each day. The DRC site accommodated this by notifying women in advance about which days to present at hospital. There were, as a result, ample patients each day of the training. In fact, the DRC site staff was remarkably capable in overcoming this concern and the many issues of intermittent power and management of time that the training presented.

The language of instruction in the DRC is French, requiring all training materials and slide presentations to be translated prior to the training. However, because Lingala is the language most commonly understood, and because much of the training was interactive, hands-on training and communication with the patient was an essential component of the training, an obstetrician from Kinshasa with strong ultrasound training skills and fluency in both languages was hired to conduct the training.

### Quality Assurance

A system of quality assurance was developed that entailed reviewing specific images from each ultrasound scan for a period of 3 months after the initial training and then 10%–20% of the ultrasound exams thereafter. These were reviewed on a website by UW radiologists and, in the case of the DRC, by the obstetrician from Kinshasa who conducted the training. In the DRC, this entailed saving the images on flash drives at the time of the scan and transporting the flash drives at least every 2 weeks to Gemena where Internet connectivity is sufficient for uploading. Remedial training that was indicated by the quality assurance review was conducted by a physician from Gemena with ultrasound imaging skills who had also served as a co-trainer during the initial 10-day course. Through email correspondence, he received instructions on which trainees needed help with which aspects of the training, a system that matched the recommendations of another study.^9^ This quality assurance system is discussed in detail elsewhere.^12^ During the course of the study, however, this local physician took a position that kept him from playing this role. The solution was to assign the role of relaying these instructions to the most skilled of the trainees as a means of remedial training, which proved to be successful.

### Health Systems Integration

The concept of screening with ultrasound requires confirmation of the findings of the screening. The training of the sonographers was comprehensive, including, as mentioned previously, an initial 2-week course in basic obstetric ultrasound screening with 3 months of quality assurance review, periodic observation, and targeted remedial training for ultrasound-naïve health personnel. However, it was never considered sufficient for the trainees to make definitive diagnoses. Patients screened by trainees require confirmatory scans by a fully trained sonographer and physician before the diagnosis of any condition is made and acted upon. The intervention, by design, engaged referral hospital personnel in this confirmatory role. At best, health centers in the Global Network clusters provided Basic EmONC. Much of the value of introducing ultrasound into the health clusters was in providing visual and metrical information to encourage women with high-risk pregnancies to seek a higher level of care.^13^ With the engagement of referral hospital personnel, the new possibility of using this information to help streamline the care patients received at referral hospitals existed.

The barriers that keep referred patients from presenting at hospitals appear to be many and are not fully understood. While studies have begun to explore these barriers in similar settings,^14^^–^^16^ the First Look study is being complemented by a qualitative survey for further understanding. From the rural patients' perspective, traveling to a city-based referral hospital without guidance can entail having to find where to receive care and to wait separately for mandatory antenatal care provision, ultrasound scanning, and care from an obstetrician. In some hospitals linked to Global Network clusters, this process can consume several days before a confirmatory ultrasound is made and an appointment with a clinician who has the results of the ultrasound exam occurs. This may be sufficiently discouraging. On the other hand, with the information from the ultrasound screening and the engagement of a sonographer and physician at the referral hospital, this process may be streamlined. Determining with the patient a date to present at the referral hospital, communicating this to the hospital, indicating where and to whom the patient should present on arrival, providing a pass that confirms this, and outlining to the patient what the visit will entail may encourage patients to overcome some of the obstacles to referral.

Providing guidance on referral and systems management was part of the study's protocol; however, the extent to which this guidance has effected change in the health systems at the 5 country sites varied in relation to the willingness of health systems to attempt the proposed changes. The Karawa health system, as mentioned previously, was included in the study in part because of its expressed willingness to participate. Moreover, the hospitals where the initial trainings took place and to which participating health centers referred high-risk pregnancies appear to have been more inclined to develop streamlining processes. These processes were built around the hospital sonographers who served as co-trainers of the field-based/health center sonographers. This was true in Guatemala and Zambia. Streamlining in Kenya and Pakistan, where the trainings occurred at hospitals not directly linked to the health centers, was more problematic.

In the DRC, training engaged a doctor and a nurse employed by Karawa Hospital who had some ultrasound imaging skills. The doctor was enthusiastic about the ultrasound intervention and played the important role of performing confirmatory scans at the hospital. Time constraints limited his ability to play an active role in tracking and receiving patients, meaning these roles were assumed by the hospital nurse who had participated in the trainings. She was significantly less interested in the training and the study, and her ultrasound skills were minimal and improved less during the training than those of the other participants. This was reflected in her lack of willingness to participate in developing and maintaining the streamlining process. Several months into the study, the doctor was transferred to another hospital and both the confirmatory process and the streamlining process were put into peril.

A solution sprang from an earlier decision to train 6 nurses for the 5 health centers. The sixth sonographer was positioned at the hospital and performed confirmatory scans in collaboration with physicians. In addition, he developed the streamlining processes and communicated with the screening nurses at the health centers about anticipated and non-compliant referrals. Moreover, because blood and anesthesia were not always readily available, this nurse helped the hospital and patients coordinate the timing and blood donors necessary for cesarean deliveries. This temporary solution aimed to simulate the structural change a health system would need to undergo were the intervention to be more permanent and integral to it.

## LOOKING FORWARD

We have discussed the challenges of implementing antenatal ultrasound screening in a low-resource setting and how these challenges were addressed during the study period. We believe our methods of addressing these challenges may help those considering research on similar interventions. The article also describes the complexities of implementing the intervention within the context of the study. In addition to the primary study, examining impact of ultrasound on mortality and morbidity, we have undertaken an economic analysis of the intervention and a qualitative survey to evaluate patient and health care personnel's perceptions of ultrasound in obstetric care and opportunities to improve that care. Both were undertaken with the aim of understanding the implications and limits of the impact of this intervention as it was designed. Combined, they aim to aid in the consideration of how ultrasound technology should be employed in low-resource settings.

### Implications for Scale-Up

While we have described the necessary prerequisites for establishing a research project focusing on providing routine ultrasound evaluations during prenatal care, we recognize additional resources were made available to the study communities through the First Look study—resources that will likely not be available to a clinical program. We demonstrated that it was feasible to conduct a technically challenging intervention even in remote and rural areas with minimal infrastructure, exemplified by our experience in Equateur Province, DRC.

However, we also recognize that multiple challenges would be faced by health systems implementing this intervention in the absence of research support. Specifically, political will, substantial resources, and continuous training and retraining would be required to initiate and maintain the ultrasound program over time. As described elsewhere, because of failure to maintain equipment and resultant breakdowns, absence of service providers, and high costs, developing-country hospitals are famous for abandoned equipment from well-intentioned donors. In order for an ultrasound program to be successful in a resource-limited setting, it would need to include continuous staff training and a system to provide quality assurance, the ultrasound equipment and its ongoing maintenance, and a continuous source of disposable supplies. These conditions do not happen by chance; they require an enabling environment as well as dedicated health care workers. Even short lapses in provider training and oversight and in the provision of supplies and equipment maintenance are sufficient to cause a program to fail.

In this light, the preliminary results from the First Look study indicate that taking routine antenatal ultrasound screening to scale is not warranted.^17^ The clinical applications of compact ultrasound equipment, on the other hand, continue to expand. Applications now include trauma imaging, focused echocardiography, obstetric emergencies, lung diseases, surgical emergencies, deep venous thrombosis, dehydration evaluation, and diagnostic breast ultrasound.^18^^–^^20^ As such, the lessons learned in implementing the First Look study should be of benefit for those considering employing the expanding uses of ultrasound or other imaging technology into low-resource settings.

Preliminary results from the First Look study indicate that taking routine antenatal ultrasound screening to scale is not warranted.

### Extrapolating From the Lessons Learned in the DRC

The process of implementing the study in Equateur Province in the DRC provides insight into where and how similar interventions might be deployed. Certain perceived thresholds below which the impact of the ultrasound intervention may be prohibitively limited become apparent. These thresholds include: (1) a functioning health system with staffed facilities providing antenatal care and assisted deliveries; (2) a referral hospital providing continuous CEmONC; (3) a distance and cost of existing means of transportation from health center communities to referral hospitals that most patients and their families can cover; (4) costs of maternal health care services that most patients can be expected to pay given the advance notice of a high-risk pregnancy that ultrasound provides.

In places that exceed such thresholds, determining the efficacy of an intervention using ultrasound or future imaging modalities and analyzing its cost relative to other interventions remain as pertinent to evaluating the need for its inclusion in low-resource settings as they were at the initiation of the First Look study. What has surfaced throughout this review is the need for structural change within health systems to accommodate the ultrasound intervention. Its importance may warrant a new way of viewing this and similar interventions. One might consider ultrasound screening as a means of strengthening referral systems. By targeting systems with an expressed willingness to take advantage of the information that ultrasound screening provides, a country could consider the provision of ultrasound as an incentive for making the necessary structural changes. For example, those systems with physicians and sonographers at referral hospitals who are willing and able to train and support health center personnel and provide a blueprint for the development of streamlining processes for referral might be awarded the opportunity to integrate ultrasound screening.

The First Look study surfaced the importance of structural change within health systems to accommodate ultrasound interventions.

Another criterion might be a plan for managing the compliance of health center personnel to integrating ultrasound screening into the provision of care. This compliance might also involve an incentive system. Using obstetrics as an example, the early detection of high-risk pregnancies coupled with the storing of a patient's images during health center screenings and confirmatory scans by referral hospital sonographers might provide the means of recording those patients that were referred and successfully delivered at referral hospitals. These records could provide the basis for incentivizing successful preventative maternal care.

Consideration of the potential structural changes to health systems made possible by the new information that ultrasound brings to low-resource settings might best serve as a starting point for conceiving future interventions. Improving the efficiency of health systems with the flow of this information may further benefit patients encouraged by this same information to seek higher levels of care.

### Future Considerations

The implementation of this ultrasound intervention in the DRC and the 4 other country sites has shown insights into which similar interventions might be targeted and how they might be structured to be most effective. Moreover, the consideration of introducing ultrasound screening as a means to strengthen referral systems has surfaced as a promising approach to improving health outcomes in rural, low-resource settings. Future initiatives for expanding the use of ultrasound in these settings may benefit from this broader consideration.

## Notes

**Peer Reviewed**
